# Evaluation of the efficacy and safety of a new flex‐rigid pleuroscope

**DOI:** 10.1111/crj.13274

**Published:** 2020-09-28

**Authors:** Satoru Ishii, Hiromu Watanabe, Manabu Suzuki, Masao Hashimoto, Motoyasu Iikura, Shinyu Izumi, Masayuki Hojo, Haruhito Sugiyama

**Affiliations:** ^1^ Department of Respiratory Medicine National Center for Global Health and Medicine Tokyo Japan; ^2^ Course of Advanced and Specialized Medicine Juntendo University Graduate School of Medicine Tokyo Japan

**Keywords:** flex‐rigid pleuroscope, LTF‐Y0032, pleural biopsy, pleural effusion

## Abstract

**Objective:**

New flex‐rigid pleuroscope enables observations with a maximum angle of curvature of 180^°^, allowing visualization of the area near the insertion site of the pleuroscope. And, it improved the image quality and channel inner diameter. The aim of this study was to evaluate the clinical effectiveness and safety of a new flex‐rigid pleuroscope.

**Methods:**

A retrospective analysis of patients who were examined with a new flex‐rigid pleuroscope under local anesthesia at our institution was conducted.

Pleuroscopy was performed in 33 patients with undiagnosed exudative pleural effusions from December 2016 to March 2019.

**Results:**

A total of 33 patients (10 women, 23 men); their median age 74 years (range 24‐90) were investigated. Pleuroscopy showed that 18 had malignant pleural disease (54%), and 15 had benign pleural diseases (46%). The top three most frequent causes of pleural disease were pleural metastases of lung carcinoma (30.3%), pyothorax (15.1%), and malignant pleural mesothelioma (12.1%). In 32 cases (97%), observation at the introducer insertion site was possible. It was not possible in one case due to hard adhesions. The diagnostic rate was 100%, and the complication rate was 6.1%. There were no major complications, and minor complications included mild pain (one case) and minor bleeding (one case) that was stanched spontaneously.

**Conclusions:**

The new flex‐rigid pleuroscope is effective and safe for diagnosing pleural effusions. The improved bending angle is likely to minimize the blind area. The new pleuroscopy fiberscope may improve the diagnostic rate.

## 1 INTRODUCTION

Even with examinations such as cytological diagnosis of specimens collected by thoracentesis, the etiology of approximately 15% of pleural effusions remains undiagnosed.^1^ Diseases such as malignant mesothelioma are difficult to diagnose. Although cytological diagnosis and blind pleural biopsy with thoracentesis are easy procedures for evaluating pleural disease, the diagnostic yields are reported to be low.^2^, ^3^ Video‐assisted thoracic surgery (VATS) provides high diagnostic yield, but the procedure requires general anesthesia and is highly invasive.^4^ Pleuroscopy under local anesthesia is less invasive, with a short examination time and more popular in Japan. With this procedure, biopsy of the parietal pleura can be carried out under direct observation, which leads to a higher diagnostic yield as compared to cytological diagnosis and blind pleural biopsy.^5^ Moreover, the complication rate is also lower, with major complications (pyothorax, pneumothorax, etc) reportedly occurring in 1.8%, and minor complications (generation of heat, sharp pain, etc) occurring in 7.3% of patients, indicating that it is a relatively safe procedure.^6^

A drawback of pleuroscopy under local anesthesia is its limited observation range in the thoracic cavity. In association with the Olympus Corporation, we developed a new pleuroscope for use under local anesthesia. The aim of this study was to evaluate the clinical effectiveness and safety of the new pleuroscope for undiagnosed pleural effusions.

## 2 MATERIALS AND METHODS

### 2.1 Patients

A single‐center retrospective analysis of patients who were examined with a new fiberscope for pleuroscopy under local anesthesia at the National Center for Global Health and Medicine was conducted. Pleuroscopy was performed in 33 patients with undiagnosed exudative pleural effusions from December 2016 to March 2019. Written informed consent was obtained from all the patients for the pleuroscopy procedure. The study protocol was approved by the Internal Review Board of our institution (NCGM‐G‐003237‐00). Patients were provided an information disclosure document explaining the study and were excluded from analysis if they opted out.

### 2.2 Pleuroscopy

**FIGURE 1:**
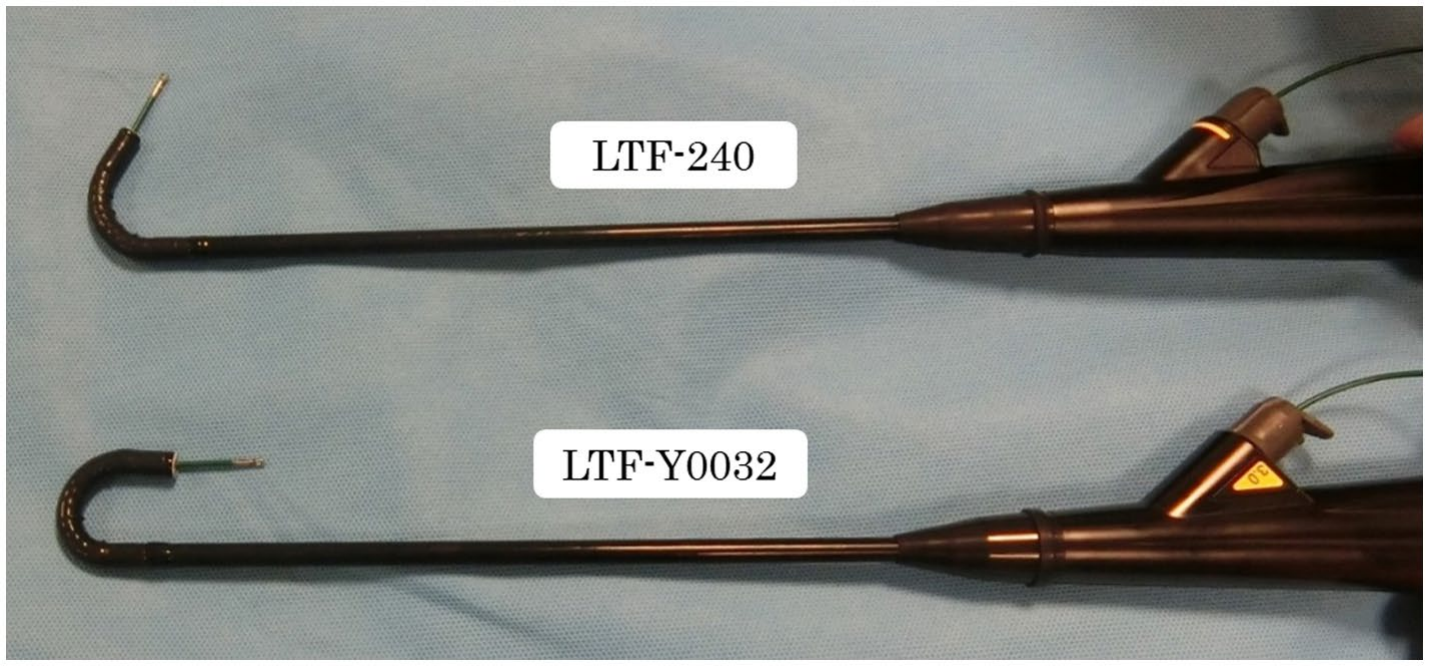
The conventional pleuroscope (LTF‐240) with the inserted biopsy forceps directed fully upward (upper). The new pleuroscope (LTF‐Y0032) with the inserted biopsy forceps directed fully upward (lower)

**FIGURE 2:**
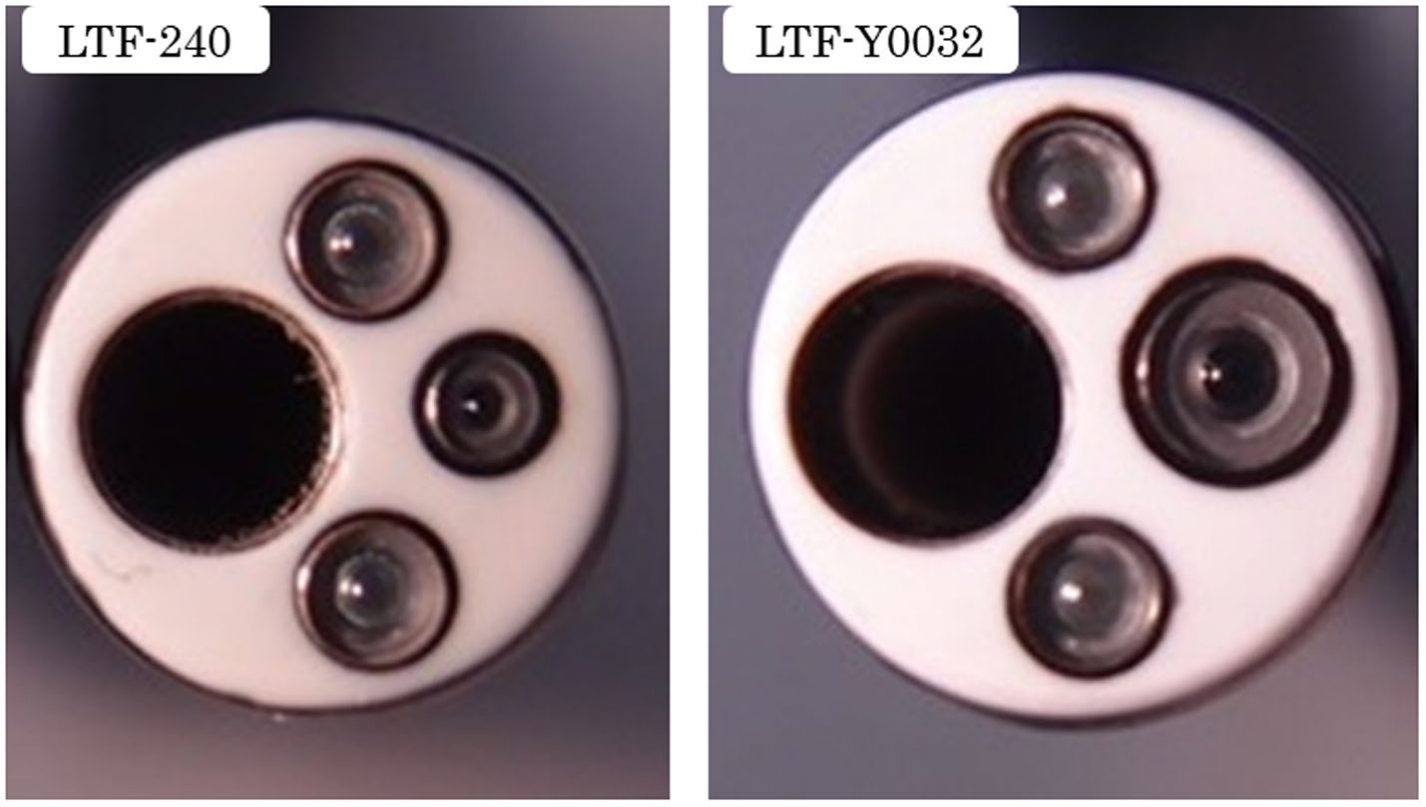
The outer diameter of the LTF‐Y0032 pleuroscope is 7.3 mm, compared with the diameter of 6.9 mm of the LTF‐240. The internal diameter of the LTF‐Y0032 is 3.0 mm, compared with 2.8mm of the LTF‐240

**FIGURE 3:**
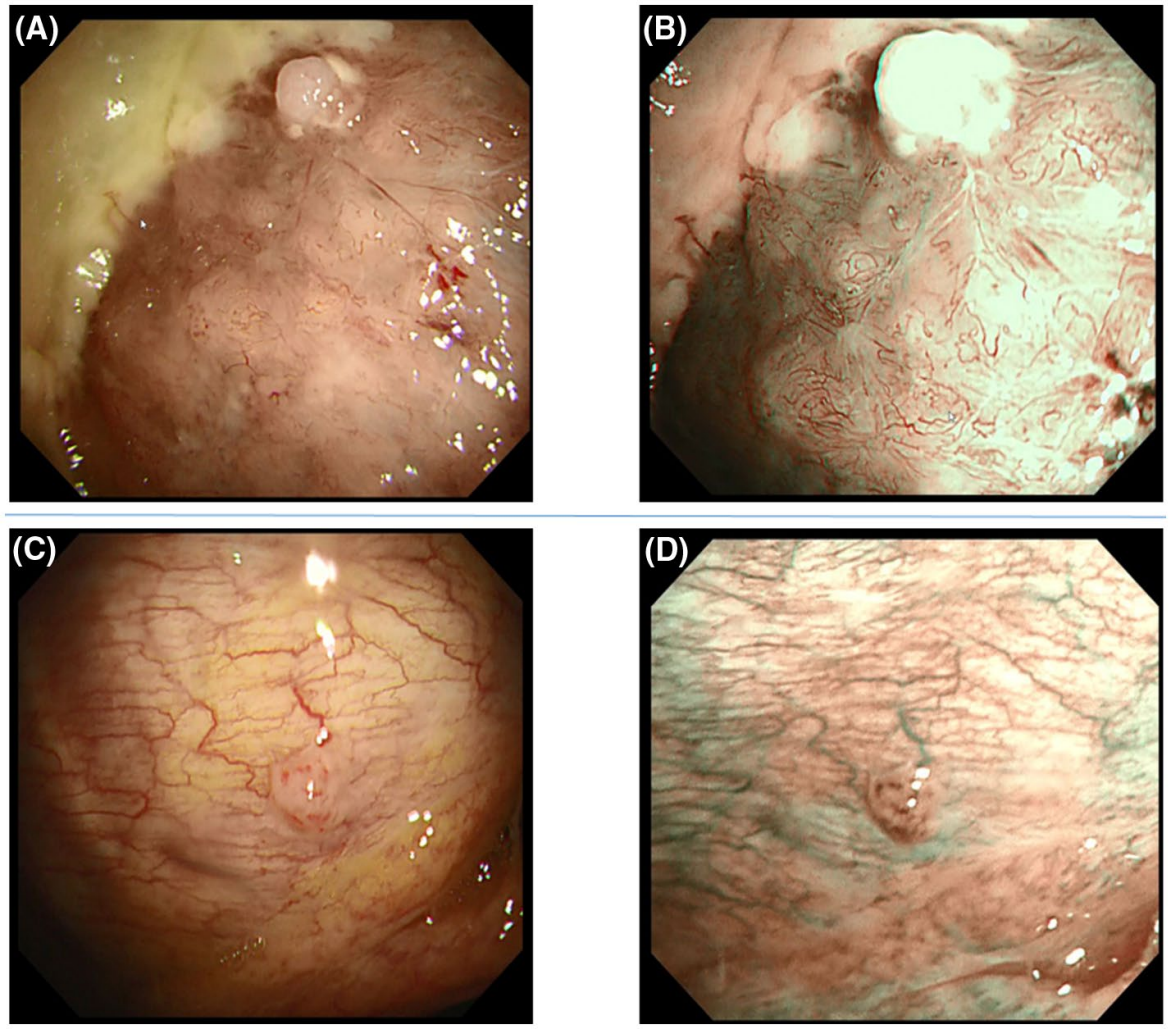
Malignant mesothelioma (epithelial type). The unevenness is clearly visible, together with the visualization of irregular blood vessels with the LTF‐Y0032. A, Narrow‐band imaging demonstrates a bosselated lesion with a network of blood vessels B, Malignant lymphoma The LTF‐Y0032 more clearly demonstrated the nodule with a network of blood vessels C, Narrow‐band imaging demonstrates the presence of meandering blood vessels on the pleura and punctate vessels on the nodule (D)

All pleuroscopic examinations were performed in the operating room. For the purpose of pain control, 15 mg of pentazocine were injected intramuscularly before the procedure. After establishment of a peripheral intravenous line, the patient was placed in the lateral decubitus position, with the side of the pleural effusion being uppermost. In all patients, chest ultrasonography was performed before pleuroscopy to evaluate the pleural effusion. Midazolam 1mg was then slowly administered intravenously for sedation. A 2‐cm incision was made just above the level of the effusion under local anesthesia with 15 to 20 mL of 1% lidocaine. The new pleuroscope was used for visualization of the thorax. The procedure was performed with a single port. The conventional pleuroscope (LTF‐240, Olympus medical systems, Tokyo, Japan) has a maximum angle of curvature of 130°and this angle is further reduced by the insertion of biopsy forceps, but the new pleuroscope (LTF‐Y0032, Olympus Medical Systems, Tokyo, Japan) enables the observations with a maximum angle of curvature of 180^°^, allowing visualization of the area near the insertion site of the pleuroscope (Figure 1). The outer diameter of the LTF‐Y0032 pleuroscope is 7.3 mm, compared with a diameter of 6.9 mm of the conventional pleuroscope. The internal diameter of the LTF‐Y0032 is 3.0mm, compared with 2.8mm of the conventional pleuroscope (Figure 2).The Olympus image quality system of the LTF‐Y0032 is BF‐H290, compared with BF‐260 of the conventional pleuroscope. The LTF‐Y0032 has a higher‐definition image than the conventional pleuroscope. Narrow‐band imaging (NBI) was used for the visualization of vascular patterns in all cases (Figure 3).After examining the thorax, approximately seven pleural biopsies were obtained. At the end of the examination, a chest tube was placed to drain the effusion.

### 2.3 Pathological diagnosis

Biopsy samples were submitted for pathology evaluation, using additional staining as was appropriate for the disease. For a diagnosis of tuberculous pleurisy, tissue samples or tissue cultures had to be positive for *M tuberculosis* by auramine staining and Ziehl‐Neelsen staining or pathological assessment had to demonstrate the presence of caseating granulomas.

## 3 RESULTS

The number of patients who underwent pleuroscopy under local anesthesia for the evaluation of undiagnosed pleural effusion from December 2016 to March 2019 was 33(10 women, 23 men), median age 74 years (range 24‐90). Pleural effusion occurred only on the left side in 13 (39%) and only on the right side in 20 (61%) patients. The proportions of small, moderate, and large size of pleural effusions were seen in 2 (6%), 19 (58%), and 12 (36%) patients, respectively. The appearance of the pleural effusion was bloody in 19 patients (58%) and yellow in 14 (42%) patients. The median examination time was 61.3 minutes (range 37‐117). Parietal pleural observations showed 18 tumors and nodules (55%), 6 pleural hypertrophy (18%), 5 adhesion (15%), and 4 pleural plaques (12%) (Table 1).

**TABLE 1:** Patient characteristics

Characteristics	
Subjects (n)	33
Age median (range) years	74 (24‐90)
Sex n (%)	
Men	23 (70)
Women	10 (30)
Side of pleural effusion n (%)	
Left side	13 (39)
Right side	20 (61)
Side of effusion n (%)	
Small	2 (6)
Moderate	19 (58)
Large	12 (36)
Effusion appearance n (%)	
Bloody	19 (58)
Yellow	14 (42)
Pleural observation n (%)	
Tumors and nodules	18 (55)
Pleural plaques	4 (12)
Pleural hypertrophy	6 (18)
Adhesion	5 (15)

**FIGURE 4:**
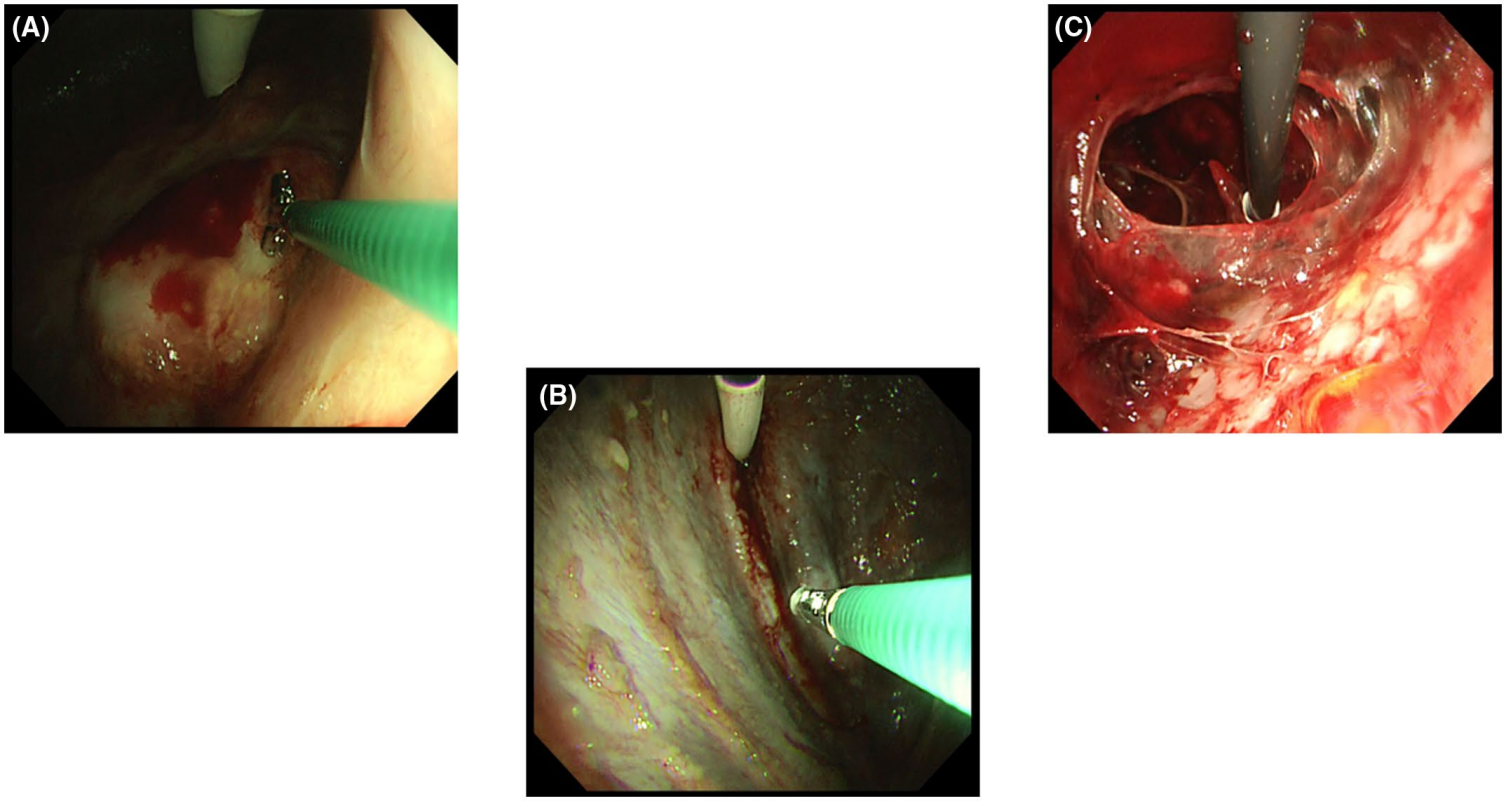
Malignant mesothelioma (biphasic type). The LTF‐Y0032 is capable of observations at a maximum curvature of 180° when directed fully upward, enabling the observation of masses at the introducer insertion site and close to the introducer. Biopsy forceps are inserted and moved near the mass, which is close to the introducer (A). Pleural metastasis of lung carcinoma Observation of many nodules around the insertion site is possible (B). Pyothorax Curettage can be performed over a wide range (C)

In 32 cases (97%), observation at the introducer insertion site was possible (Figure 4). It was not possible in one case due to hard adhesions.

On pathology examination, there were 18 malignant tumors and 15 benign diseases. The malignant tumors included 10 pleural metastases of lung carcinoma (30.3%), 4 malignant pleural mesotheliomas (12.1%), 3 malignant lymphomas (9.2%), and 1 pleomorphic carcinoma (3%). Benign diseases included five pyothorax (15.1%), four nonspecific pleurisy (12.1%), three tuberculous pleurisy (9.1%), two asbestos‐related pleurisy (6.1%), and one IgG‐4 related disease (3%) (Table 2). The diagnostic rate was 100%. We see the progress of nonspecific pleurisy for more than half a year, but no re‐exacerbation of pleural effusion has been observed.

**TABLE 2:** Causes of pleural effusions

Cause	n ( %)
Malignancies	Pleural metastasis of lung carcinoma 10 cases (30.3%)
	Malignant pleural mesothelioma 4 cases (12.1%)
	Malignant lymphoma 3 cases (9.2%)
	Pleomorphic carcinoma 1 case (3%)
Benign diseases	Pyothorax 5 cases (15.1%)
	Nonspecific pleurisy 4 cases (12.1%)
	Tuberculous pleurisy 3 cases (9.1%)
	Asbestos‐related pleurisy 2 cases (6.1%)
	IgG4‐related disease 1 case (3%)
Diagnostic rate	100%

The complication rate was 6.1%. No serious adverse events were observed. Minor complications included mild pain in one case and mild bleeding in one case, but it was stanched spontaneously (Table 3).

**TABLE 3:** Complications of pleuroscopy

Complication	Cases
Major complication	None
Minor complication	
Mild Pain	1 case
Minor bleeding	1 case
Complication rate	6.1%

## 4 DISCUSSION

In this study, the new pleuroscope was found to be effective and safe for undiagnosed pleural effusions. To the best of our knowledge, this is the first original report of a new pleuroscope. The new pleuroscope has an improved bending angle, image quality, and channel inner diameter.^7^ The improved bending angle is likely to minimize the blind area.

VATS is known to have a high diagnostic yield for pleural diseases, but with the risks associated with general anesthesia and the invasiveness of the procedure.^8^

VATS can be performed with two ports, while pleuroscopy under local anesthesia can be performed with a single port. Pleuroscopy under local anesthesia is less invasive and has been frequently performed by pulmonologists in recent years.^6^ McDonald et al reported that pleuroscopy under local anesthesia and VATS have similar diagnostic yields and safety in patients with undiagnosed pleural effusions. The VATS procedure‐related average cost was Canadian $7962 higher than the $2815 for pleuroscopy under local anesthesia.^9^

This procedure enables direct visualization of the parietal pleura, while biopsy allows for safe and accurate diagnosis of pleural diseases. Although cytological diagnosis is common for carcinomatous pleurisy, the positive cytology ratio is reportedly only 62%, the positivity rate of a blind pleural biopsy is 42%, and the diagnostic yield of the combination of both reaches 74% at best.^10^ The diagnostic yields with pleuroscopy under local anesthesia, moreover, have been reported to be as high as 79 ~ 96%.^11^, ^12^ The reason that the diagnosis rate was not 100% was blind spots. The conventional pleuroscope has a limited bending angle, resulting in blind spots. The pleuroscope is flexible only at the tip; if it is advanced forward with the tip bent, it will leave the biopsy site. In this case, one can loosen the curve, tilt the pleuroscope slightly and advance it to the target, and then, apply the curve again. However if the pleuroscope is tilted too much, the ribs can be damaged, so care must be taken and technical skill is required. The new pleuroscope bends up to 180 degrees, so a biopsy can be performed using the curvature of the tip without tilting. In addition, with a high‐definition image, the possibility of missing small lesions is reduced. For this reason, there is a high possibility that the diagnosis rate is further improved with the new pleuroscope. Image quality with the new pleuroscope was also clearer with NBI than with white light imaging, allowing easy identification of irregular vascularity. Switching from white light to NBI involves the mere press of a button on the pleuroscope, making it very simple, useful, and non‐invasive for patients. NBI is reportedly useful for visualization of vascular patterns; irregular vascularity on NBI was more often seen in malignant diseases than in benign diseases.^13^ The diagnostic rate was 100%. Four cases of malignant pleural mesothelioma had a mass or nodule of the parietal pleura, and biopsies were easy in the present study. However, flat‐type lesions of malignant pleural mesothelioma cannot be diagnosed with conventional biopsy forceps.^14^ If we had the flat type of mesothelioma, the diagnostic rate would be decreased. It is necessary to collect not only the pleural surface but also the subpleural tissue. An increasing number of facilities use a cryoprobe when performing pleuroscopy under local anesthesia.^15^, ^16^A cryoprobe is useful to obtain sufficient tissues for flat‐type lesions and solid masses. The internal diameter of the new pleuroscope is 3.0mm, compared with the diameter of 2.8mm of the conventional one, making it easier to operate the cryoprobe.

Pleural metastasis of lung carcinoma is the most common of pleural disease. Tissue sampling could originally only establish the diagnosis of lung cancer, but now sufficient tissue sampling is needed for next generation sequencing for epidermal growth factor receptor (EGFR), anaplastic lymphoma kinase (ALK), ‐etc‐. From one tissue sample, comprehensive cancer genetic panel testing (CGP) involving multiple genetic tests at the same time is done in Japan.^17^ These CGP assays generally require a minimum tumor cell content of 20% to 30% to have adequate sensitivity for all classes of alterations.^18^, ^19^A larger tumor cell size improves the overall impression of the tumor cell content. Pleural cryobiopsy is consistently yields larger tissue specimens than flexible forceps biopsy. In addition, cryobiopsy results in fewer instances of crushed tissue than forceps biopsy. However, the rate of clinically relevant bleeding was higher after cryobiopsy procedures than after forceps biopsy.^20^ Therefore, the decision to use cryobiopsy or forceps biopsy must be made based on clinical and thoracoscopic findings.

The results of the present study must be considered in light of several limitations. First, this study was a retrospective review of medical records with a small sample size. Second, the diagnostic rate would have been lower if there had been solid tumors or flat type mesotheliomas. Third, as confirmation of the operability of new pleuroscope and medical systems was performed, the procedures took longer to complete than usual. Fourth, although improvements to the new pleuroscope were made at various points, it continues to have limited operability compared with VATS. Specifically, VATS is superior for treating pyothorax than pleuroscopy under local anesthesia. Therefore, pleuroscopy under local anesthesia should be used only for diagnostic purposes and the treatment of mild pyothorax.

In conclusion, the new pleuroscope has an improved bending angle, image quality, and channel inner diameter. The improved bending angle is likely to minimize the blind area. The new pleuroscope can be operated safely and may improve the diagnostic rate.

## CONFLICTS OF INTEREST

Olympus lent the authors department the LTF‐Y0032 pleuroscope and CV‐290 endoscopy system. The authors have stated explicitly that there are no conflicts of interest in connection with this article, respectively.

## AUTHOR CONTRIBUTIONS

*Substantial contributions to the design of the work, the acquisition, analysis or interpretation of date, drafting and revising the manuscript critically for important*: Ishii

*Substantial contributions to the design of the work, the acquisition, analysis or interpretation of date, drafting and revising the manuscript critically for important, final approval of the version to be published*: Watanabe, Suzuki, Hashimoto, Iikura, Izumi, Hojo

*Substantial contributions to the design of the work, the acquisition, analysis or interpretation of date, drafting and revising the manuscript critically for important, final approval of the version to be published, revising the manuscript for important intellectual content*: Sugiyama

## ETHICS

The study protocol was approved by the Internal Review Board of our institution (NCGM‐G‐003237‐00).
