# Corrigendum

**DOI:** 10.1111/crj.13550

**Published:** 2022-12-02

Ishii S, Watanabe H, Suzuki M, Hashimoto M, Iikura M, Izumi S, Hojo M, Sugiyama H. Evaluation of the efficacy and safety of a new flex‐rigid pleuroscope. *The Clinical Respiratory Journal*. 2021; 15:91–96.

In the above article, author Tetsuo Hara has been added as a new author.

The new order of author should be as follows:

Satoru Ishii^1,2^, Hiromu Watanabe^1^, Manabu Suzuki^1^, Masao Hashimoto^1^, Motoyasu Iikura^1^, Shinyu Izumi^1^, Masayuki Hojo^1^, Tetsuo Hara^2^, Haruhito Sugiyama^1^

The authors apologize for these errors.

